# Complete Genome Sequence of Klebsiella pneumoniae Podophage Patroon

**DOI:** 10.1128/MRA.00461-19

**Published:** 2019-05-23

**Authors:** Rainie Tran, Rohit Kongari, Lauren Lessor, Jason J. Gill, Mei Liu

**Affiliations:** a Center for Phage Technology, Texas A&M University, College Station, Texas, USA; Queens College

## Abstract

Klebsiella pneumoniae infection is a serious concern in hospital settings due to the continuing emergence of multidrug-resistant strains. The study of *K. pneumoniae* phages may help the development of new treatment strategies. Here, the complete genome sequence of K. pneumoniae phage Patroon, a T3/T7-like phage, is presented.

## Abstract (precis)

Klebsiella pneumoniae infection is a serious concern in hospital settings due to the continuing emergence of multidrug-resistant strains. The study of *K. pneumoniae* phages may help the development of new treatment strategies.

## ANNOUNCEMENT

Klebsiella pneumoniae is a Gram-negative bacterium well known as an opportunistic pathogen that causes pneumonia, septicemia, and urinary tract infection (1, 2). K. pneumoniae infection is a serious concern in hospital settings due to the continuing emergence of multidrug-resistant strains carrying the *bla*_KPC_ gene (3). The study of *K. pneumoniae* phages may help us develop new treatment strategies.

Phage Patroon was isolated from influent water from the municipal wastewater treatment plant in Bryan, TX, in 2016, using a carbapenem-resistant (KPC^+^) K. pneumoniae clinical isolate of sequence type 258 as the host. Host bacteria were cultured on tryptic soy broth or agar (Difco) at 37°C with aeration. Phages were isolated and propagated by the soft agar overlay method (4). Phage genomic DNA was prepared using a modified Promega Wizard DNA cleanup kit protocol, as described previously (5). Pooled indexed DNA libraries were prepared using the Illumina TruSeq Nano low-throughput (LT) kit, and the sequence was obtained from the Illumina MiSeq platform using the MiSeq V2 500-cycle reagent kit, following manufacturer’s instructions, producing 667,982 paired-end reads for the index containing the phage genome. The quality of the reads was checked in FastQC 0.11.5 (https://www.bioinformatics.babraham.ac.uk/projects/fastqc/), trimmed with FastX-Toolkit 0.0.14 (http://hannonlab.cshl.edu/fastx_toolkit/download.html), and assembled in SPAdes 3.5.0 (6). The assembled genome was closed with PCR using primers (5′-GCTGGTAAGGAAGTCGGTAAA-3′, 5′-GTCGTTAGTTAGGCGGTCATAG-3′) facing off the ends of the assembled contig and Sanger sequencing of the resulting product, with the contig sequence manually corrected to match the resulting Sanger sequencing read. Protein-coding genes were predicted using GLIMMER 3.0 (7) and MetaGeneAnnotator 1.0 (8) and corrected manually if needed. The tRNA genes were predicted using ARAGORN 2.36 (9). Protein functions were predicted by comparing the sequence homology to proteins found using BLASTp 2.2.28 (10), and conserved domains were analyzed using InterProScan 5.15-5.40 (11). All the analyses were performed under default settings using the CPT Galaxy (12) and Web Apollo (13) interfaces (cpt.tamu.edu).

The Patroon genome was assembled into a complete contig of 39,442 bp at 596.8-fold coverage. It contains 51 predicted coding sequences, with a coding density of 89% and a GC content of 50.54%. At the DNA level, Patroon is most similar (87% to 88%) to other T7-like enterobacterial phages, such as *Escherichia* coliphage ECA2 (GenBank accession number KX130726), *Yersinia* sp. phage phiYeO3-12 (GenBank accession number AJ251805), and *Salmonella* sp. phage phiSG-JL2 (GenBank accession number EU547803), as determined by the progressiveMauve algorithm (14). Patroon is a T3/T7-like phage with 47 and 45 Patroon proteins matching phages T3 and T7, respectively, determined by BLASTp (E value < 0.001). Genes encoding proteins related to phage morphogenesis, DNA replication, and recombination were identified. The lysis proteins identified consisted of a class II holin, an amidase endolysin, and an embedded i-spanin and o-spanin pair. The phage Patroon tail fiber gp44 (GenBank accession number QBQ72909) is closely related at its N terminus to other T7-like tail fibers, including the phage T7 tail fiber gp17. The C-terminal receptor-binding domain is related to only a few other phage tail fibers based on BLASTp alignment, including those of coliphage ECA2 (GenBank accession number ANN86232) and *Yersinia* sp. phage phiYeO3-12 (GenBank accession number NP_052117).

### Data availability.

The genome sequence of phage Patroon was submitted to GenBank under accession number MK608335. The associated BioProject, SRA, and BioSample accession numbers are PRJNA222858, SRR8788210, and SAMN11259695, respectively.

## References

[1] Podschun R, Ullmann U 1998 Klebsiella spp. as nosocomial pathogens: epidemiology, taxonomy, typing methods, and pathogenicity factors. Clin Microbiol Rev 11:589–603. doi:10.1128/CMR.11.4.589. 9767057 PMC88898

[2] Struve C, Krogfelt KA 2004 Pathogenic potential of environmental Klebsiella pneumoniae isolates. Environ Microbiol 6:584–590. doi:10.1111/j.1462-2920.2004.00590.x. 15142246

[3] Arnold RS, Thom KA, Sharma S, Phillips M, Kristie Johnson J, Morgan DJ 2011 Emergence of Klebsiella pneumoniae carbapenemase-producing bacteria. South Med J 104:40–45. doi:10.1097/SMJ.0b013e3181fd7d5a. 21119555 PMC3075864

[4] Adams MK 1959 Bacteriophages. Interscience Publishers, Inc, New York, NY.

[5] Summer EJ 2009 Preparation of a phage DNA fragment library for whole genome shotgun sequencing. Methods Mol Biol 502:27–46. doi:10.1007/978-1-60327-565-1_4. 19082550

[6] Bankevich A, Nurk S, Antipov D, Gurevich AA, Dvorkin M, Kulikov AS, Lesin VM, Nikolenko SI, Pham S, Prjibelski AD, Pyshkin AV, Sirotkin AV, Vyahhi N, Tesler G, Alekseyev MA, Pevzner PA 2012 SPAdes: a new genome assembly algorithm and its applications to single-cell sequencing. J Comput Biol 19:455–477. doi:10.1089/cmb.2012.0021. 22506599 PMC3342519

[7] Delcher AL, Harmon D, Kasif S, White O, Salzberg SL 1999 Improved microbial gene identification with GLIMMER. Nucleic Acids Res 27:4636–4641. doi:10.1093/nar/27.23.4636. 10556321 PMC148753

[8] Noguchi H, Taniguchi T, Itoh T 2008 MetaGeneAnnotator: detecting species-specific patterns of ribosomal binding site for precise gene prediction in anonymous prokaryotic and phage genomes. DNA Res 15:387–396. doi:10.1093/dnares/dsn027. 18940874 PMC2608843

[9] Laslett D, Canback B 2004 ARAGORN, a program to detect tRNA genes and tmRNA genes in nucleotide sequences. Nucleic Acids Res 32:11–16. doi:10.1093/nar/gkh152. 14704338 PMC373265

[10] Camacho C, Coulouris G, Avagyan V, Ma N, Papadopoulos J, Bealer K, Madden TL 2009 BLAST+: architecture and applications. BMC Bioinformatics 10:421. doi:10.1186/1471-2105-10-421. 20003500 PMC2803857

[11] Jones P, Binns D, Chang HY, Fraser M, Li W, McAnulla C, McWilliam H, Maslen J, Mitchell A, Nuka G, Pesseat S, Quinn AF, Sangrador-Vegas A, Scheremetjew M, Yong SY, Lopez R, Hunter S 2014 InterProScan 5: genome-scale protein function classification. Bioinformatics 30:1236–1240. doi:10.1093/bioinformatics/btu031. 24451626 PMC3998142

[12] Cock PJ, Gruning BA, Paszkiewicz K, Pritchard L 2013 Galaxy tools and workflows for sequence analysis with applications in molecular plant pathology. PeerJ 1:e167. doi:10.7717/peerj.167. 24109552 PMC3792188

[13] Lee E, Helt GA, Reese JT, Munoz-Torres MC, Childers CP, Buels RM, Stein L, Holmes IH, Elsik CG, Lewis SE 2013 Web Apollo: a Web-based genomic annotation editing platform. Genome Biol 14:R93. doi:10.1186/gb-2013-14-8-r93. 24000942 PMC4053811

[14] Darling AE, Mau B, Perna NT 2010 progressiveMauve: multiple genome alignment with gene gain, loss and rearrangement. PLoS One 5:e11147. doi:10.1371/journal.pone.0011147. 20593022 PMC2892488

